# A Forward Chemical Screen in Zebrafish Identifies a Retinoic Acid Derivative with Receptor Specificity

**DOI:** 10.1371/journal.pone.0010004

**Published:** 2010-04-02

**Authors:** Bhaskar C. Das, Kellie McCartin, Ting-Chun Liu, Randall T. Peterson, Todd Evans

**Affiliations:** 1 Department of Developmental and Molecular Biology, Albert Einstein College of Medicine, New York, New York, United States of America; 2 Department of Nuclear Medicine, Albert Einstein College of Medicine, New York, New York, United States of America; 3 Department of Surgery, Weill Cornell Medical College, Cornell University, New York, New York, United States of America; 4 Cardiovascular Research Center, Massachusetts General Hospital, Harvard Medical School, Charlestown, Massachusetts, United States of America; Katholieke Universiteit Leuven, Belgium

## Abstract

**Background:**

Retinoids regulate key developmental pathways throughout life, and have potential uses for differentiation therapy. It should be possible to identify novel retinoids by coupling new chemical reactions with screens using the zebrafish embryonic model.

**Principal Findings:**

We synthesized novel retinoid analogues and derivatives by amide coupling, obtaining 80–92% yields. A small library of these compounds was screened for bioactivity in living zebrafish embryos. We found that several structurally related compounds significantly affect development. Distinct phenotypes are generated depending on time of exposure, and we characterize one compound (BT10) that produces specific cardiovascular defects when added 1 day post fertilization. When compared to retinoic acid (ATRA), BT10 shows similar but not identical changes in the expression pattern of embryonic genes that are known targets of the retinoid pathway. Reporter assays determined that BT10 interacts with all three RAR receptor sub-types, but has no activity for RXR receptors, at all concentrations tested.

**Conclusions:**

Our screen has identified a novel retinoid with specificity for retinoid receptors. This lead compound may be useful for manipulating components of retinoid signaling networks, and may be further derivatized for enhanced activity.

## Introduction

Chemical genetics is an approach for identifying small molecules that alter the function of biological pathways, resulting in the induction or rescue of a specific phenotype [1], [2], [3], [4]. Forward chemical genetics involves screening a library of compounds to find small molecules that generate a consistent phenotype in a biological assay, analogous to more traditional genetic screens. An advantage of chemical genetics over traditional genetics is the inherent conditional effect, and if it relates to human disease, a small molecule may serve as a starting point for drug discovery. The ability to modulate function specifically and rapidly makes small molecules especially useful tools for studying processes like development in which the timing of protein function is critical. Currently, systematic methods for identifying small molecules that alter specific developmental processes are limited, so the number of useful small molecule developmental probes remains small. However, using combinatorial techniques, it is possible to rapidly screen a large number of small molecules and identify those that induce a novel phenotype in a cellular or embryonic system [5]. Here we describe a chemical genetic approach by interfacing a library of small molecules with developing embryos. Specifically, we are interested in characterizing new compounds that interact with developmentally important receptor-mediated pathways, acting as antagonist or agonist, in order to manipulate signaling pathways and to identify new gene targets of these pathways. For this purpose we have synthesized retinoic acid analogues and derivatives and tested their bioactivity in the context of developing zebrafish embryos.

The zebrafish is a vertebrate with tissues and organ systems that develop and are organized similarly to humans, at the anatomical, physiological and molecular levels, thereby facilitating the study of biological processes and the development of human disease models that are inaccessible or limited using invertebrate model organisms [6]. Several hundred synchronously developing embryos can be obtained from mating a single pair of adults. The embryos develop externally and it is feasible to screen large numbers of compounds in a high-throughput manner [7]. Finally, the zebrafish embryo is transparent. Therefore, internal organs and structures can be visualized in the intact, living organism, facilitating multiple or continuous observations of dynamic processes.

Retinoic acid (RA), a member of the retinoid family of lipids and mediator of vitamin A activity, is an essential morphogen in vertebrate development [8]. The formation of the body axes and the development of a number of organ systems including retina, brain, heart, the urogenital system, and lungs are dependent on RA. The developmental defects resulting from vitamin A deficiency as well as the teratogenic effects of retinoid overdose are well documented [9]. Due to its effect on cell differentiation and proliferation, RA is now being used as a therapeutic tool in dermatology and oncology [10]. For example, RA provides a paradigm for differentiation therapy, an approach to treat malignant cells that are blocked for differentiation, so that they overcome the block and resume the process of differentiation into mature cells., However, apart from success in differentiation therapy for acute promyelocytic leukemia, retinoid therapies have not translated well to other cancers. While isotretinoin, a 13-cis-retinoic acid, has been widely used for a range of dermatological conditions, it also has severe teratogenic effects including craniofacial, cardiovascular, thymic and central nervous system malformations [11]. Therefore, the identification of retinoids with novel bioactivity is of clinical relevance [12]. The most important mechanism of RA activity is the regulation of gene expression. This is accomplished by its binding to nuclear retinoid receptors that are ligand-activated transcription factors [13]. Thus, RA acts as a transcriptional activator for a large number of other downstream regulatory molecules, including enzymes, transcription factors, cytokines, and cytokine receptors. Although the overwhelming majority of experimental studies have focused on RA receptor–dependent mechanisms, there have been indications of other, non-classical modes of action. This might occur through ligand interactions with enzymes such as protein kinase C, by direct activation of electrical synapses, or from interaction with putative cell surface receptors [14]. Toward a long-term goal of identifying new antagonists and agonists that target specific pathways, we focused on retinoid signaling, since it plays key roles in patterning the body axis, in the formation of many organs, and in maintaining tissue homeostasis.

## Results

### Derivation of a library of retinoid analogues

**Figure 1:**
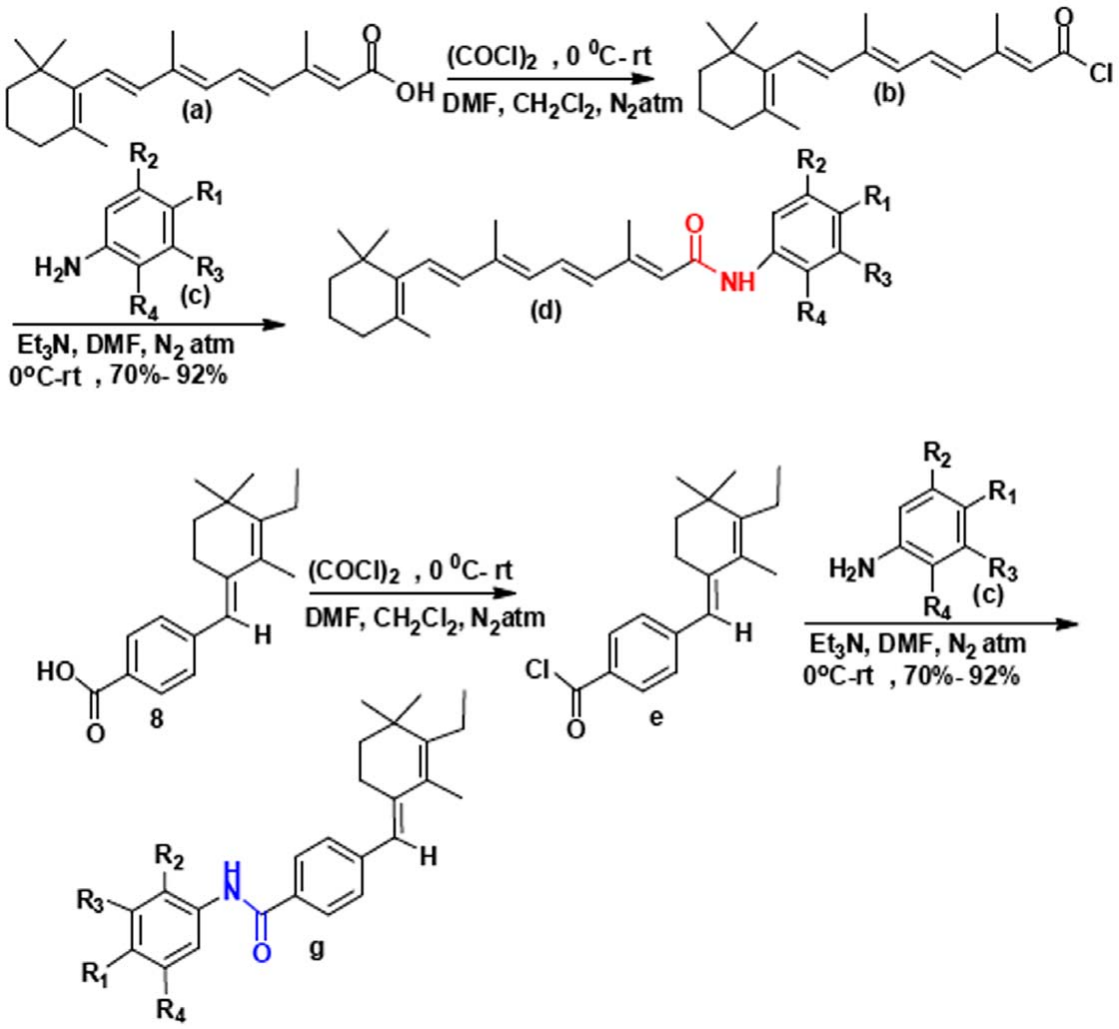
Strategy to synthesize novel retinoids. See text and Methods for details.

**Figure 2:**
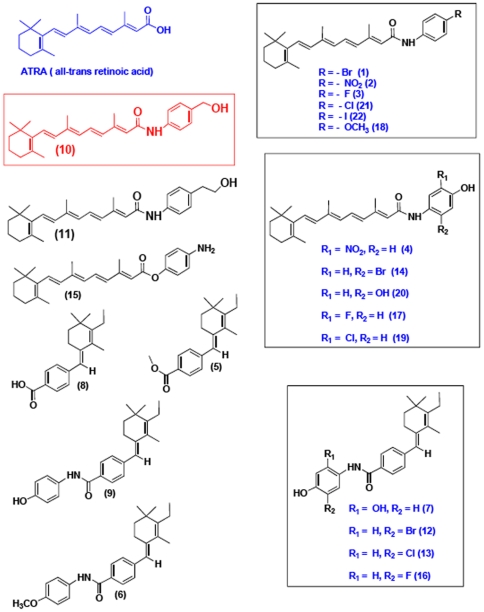
Compounds tested for activity altering normal zebrafish development. Note that compounds 10, 11, and 20 were found to generate similar phenotypes, while compound 10 (red) was chosen for further study, since it had highest activity based on a dose response study. For simplicity, highly related compounds are grouped on the right.

We first synthesized retinoic acid analogues of amides (retinoids) by coupling RA with amine derivatives (Fig. 1). First, retinoic acid was converted to retinoyl chloride, then in the presence of DMF, base, and aniline derivatives, we synthesized the corresponding 4-hydroxy phenyl amine derivatives (1, 2, 3, 4, 10, 11, 14, 15, 17, 18. 19, 20, 21 and 22). This procedure gave 80–90% yield. To develop efficacy and potency we synthesized novel retinoic acid analogue 8 [15] and then further derivatized to corresponding amide derivatives (5, 6, 7, 9, 12, 13 and 16) by amide coupling reactions. According to this strategy we purified sufficient materials to generate a small library of these retinoid derivatives (Fig. 2) to screen for bioactivity in zebrafish embryos.

### A developmental screen for analogue activity

After synthesis and purification of the compounds, they were screened for bioactivity using the zebrafish model. For this purpose, fertilized eggs were obtained from paired adults and cultured until 3–5 hours post-fertilization (hpf). Embryos were then grouped into individual wells of a 12 well plate, each well containing 3–10 embryos in 1 ml of 1X E3 buffer, 1% DMSO, and 1 mM Tris pH 7.5. Each test compound was diluted in DMSO to 10 mM stocks, and added to the cultured embryos at a final concentration of 10 µM. Treated embryos were cultured until 24 hpf and assessed for morphological alterations compared to embryos treated with vehicle (DMSO) alone. In several independent experiments the effect of compounds was consistent and penetrant. Several of the compounds (10, 11, and 20) cause similar and very significant disruptions of the body axis (not shown). Dilutions of these three compounds were compared to evaluate their relative dose-response activities, and compound 10 was found to be most active, since it altered body axis formation when added at a concentration as low as 312 nM, a concentration at which the other two compounds are not active. We designated this lead compound BT10 and characterized its activity in more detail.

**Figure 3:**
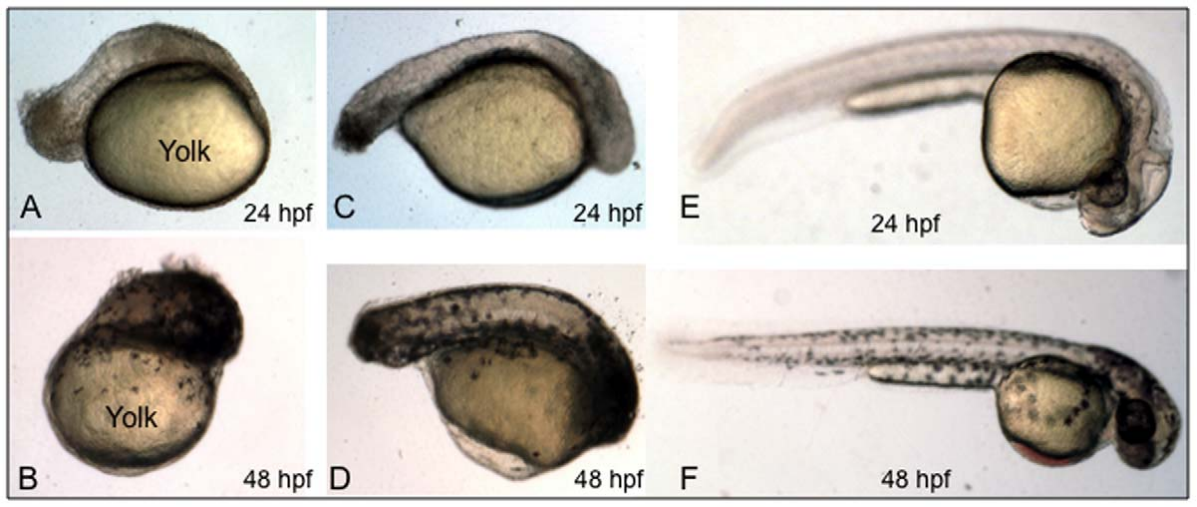
BT10 added during early embryogenesis causes a major disruption of the body axis. Shown are representative embryos (from 3 experiments, for each sample n = 20) treated with BT10 at 10 µM (A-D) compared to embryos treated only with vehicle (DMSO) as control. BT10 was added at 5 hpf (A, B) or 8 hpf (C, D). DMSO alone was added at 5 hpf (E, F). Embryos were allowed to develop until 24 hpf (A, C, E) or 48 hpf (B, D, F). The position of the yolk is indicated in panels A and B. All embryos are viewed laterally, anterior to the right, dorsal at the top.

As shown in Fig. 3A, when BT10 is added to embryos at 5 hpf there is by 24 hpf a severe truncation of the body axis, although the embryo survives and forms a simple mass of pigmented tissue on the remaining yolk by 48 hpf (Fig. 3B). We do not believe that the effect is toxic, because the embryos are alive (even moving or ”twitching“, and continuing to develop pigmentation), although the ”posteriorization“ of the embryonic trunk is severe. If addition of the compound is delayed until 8 hpf, there is now a significant increase in development of the trunk, although head structures are largely undefined and the tail is much shorter than in control embryos at 24 hpf (Fig. 3C) or 48 hpf (Fig. 3D). These embryonic ”posteriorization“ phenotypes are consistent and similar to defects caused by addition of RA [16], [17] and known RAR activators, such as DTAB [18].

### Exposure to BT10 after 24 hpf disrupts cardiovascular development

**Figure 4:**
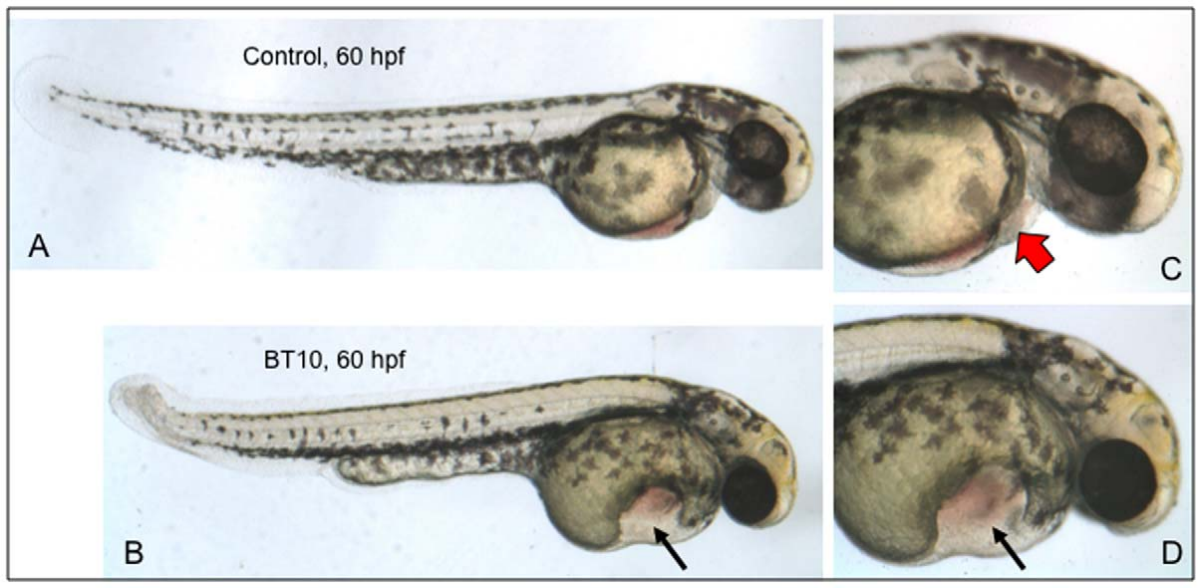
BT10 causes specific cardiovascular defects when added at 24 hpf. Shown are representative embryos (from 3 experiments, for each sample n = 20) that were cultured between 24–60 hpf in the presence of DMSO alone (A, control) or BT10 (B). Note the defined morphological structure of the ventral yolk sac (arrow). A normal looping heart tube fails to develop in these embryos. Panels C and D show higher magnification views of representative control (C) and BT10-treated (D) embryos, respectively. The red block arrow indicates blood flowing through the normal control heart, which is not evident in the BT10-treated embryos. Views are lateral, anterior to the right, dorsal at the top.

A major advantage of using small molecules for manipulating pathways is that the activity can be tightly controlled, simply by the timing of delivery. We next sought to determine if BT10 remains active and is able to disrupt later stages of development. For this purpose, embryos were cultured with BT10 (or control vehicle) added at 24 hpf, and phenotypes were evaluated at 60 hpf. In multiple, independent experiments, BT10-treated embryos display a consistent and penetrant phenotype (n>50), comprising a characteristic malformation of the ventral yolk structure and disruption of normal heart tube development (Fig. 4). While the embryos show a minor posterior tail defect, the body trunk and gross head/brain structure are relatively normal. However, the heart tube that forms is thin, non-looping, and very weakly beating. This phenotype is also consistent with those reported for altered retinoid signaling, for example due to mutation in the *aldh1a2* gene [17].

**Figure 5:**
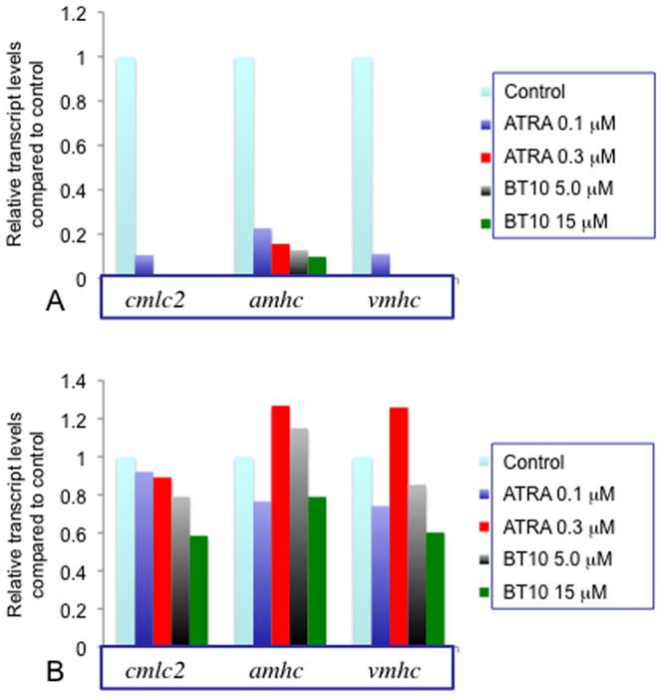
BT10, similar to RA, inhibits cardiomyocyte differentiation when added early during embryogenesis. (A) Embryos were cultured starting at 5 hpf in the presence of vehicle alone (DMSO, control), or “low” (0.1 µM, 5.0 µM) or “high” (0.3 µM, 15 µM) concentrations of ATRA or BT10, respectively. RNA was harvested at 24 hpf and used to measure by qPCR relative transcript levels, as indicated. All cardiac markers are significantly reduced in transcript levels by ATRA or BT10, and for both compounds, the ventricular marker is relatively more sensitive. (B) Embryos were treated in the same manner starting at 24 hfp, and RNA was harvested at 48 hpf. Under these conditions there was not a consistent significant inhibition of cardiomyocyte differentiation, based on levels of the same cardiac markers. These data are from one representative experiment, showing the mean relative transcript levels (normalized to the control) of triplicate qPCR assays, using 30 embryos per sample.

Previous studies showed that excess RA eliminates or sharply reduces the amount of cardiac tissue in *Xenopus* [19] or zebrafish [20] embryos. More recent studies confirmed that heart tissue is severely diminished in zebrafish embryos treated with RA at a relatively high concentration (0.3 µM). At a relatively lower concentration (0.1 µM), ventricular tissue is preferentially sensitive to RA compared to atrial tissue [21]. Therefore, we next compared the effects of adding BT10 and RA during embryogenesis on cardiac development. For this purpose we compared the effect of RA at concentrations of BT10 that generates grossly similar body axis defects (low: 5 µM BT10, 0.1 µM ATRA; high: 15 µM BT10, 0.3 µM ATRA). We used quantitative RT-PCR (qPCR) to evaluate the effect of a pan-cardiomyocyte marker (*cmlc2*) in addition to markers specific to ventricular cardiomycoytes (*vmhc*) and atrial cardiomyocytes (*ahmc*). As shown in Fig. 5A, when assayed at 24 hpf, both compounds severely diminish expression levels of cardiac markers when added at 5 hpf. Notably, similar to ATRA, embryos treated with BT10 retain detectable (albeit substantially decreased) levels of the atrial marker, under conditions that eliminate detectable expression of the ventricular marker. When added at 24 hpf, the expression levels for these cardiac markers are much less disturbed (Fig. 5B), consistent with a morphogenetic defect rather than a failure to develop cardiomyocytes.

**Figure 6:**
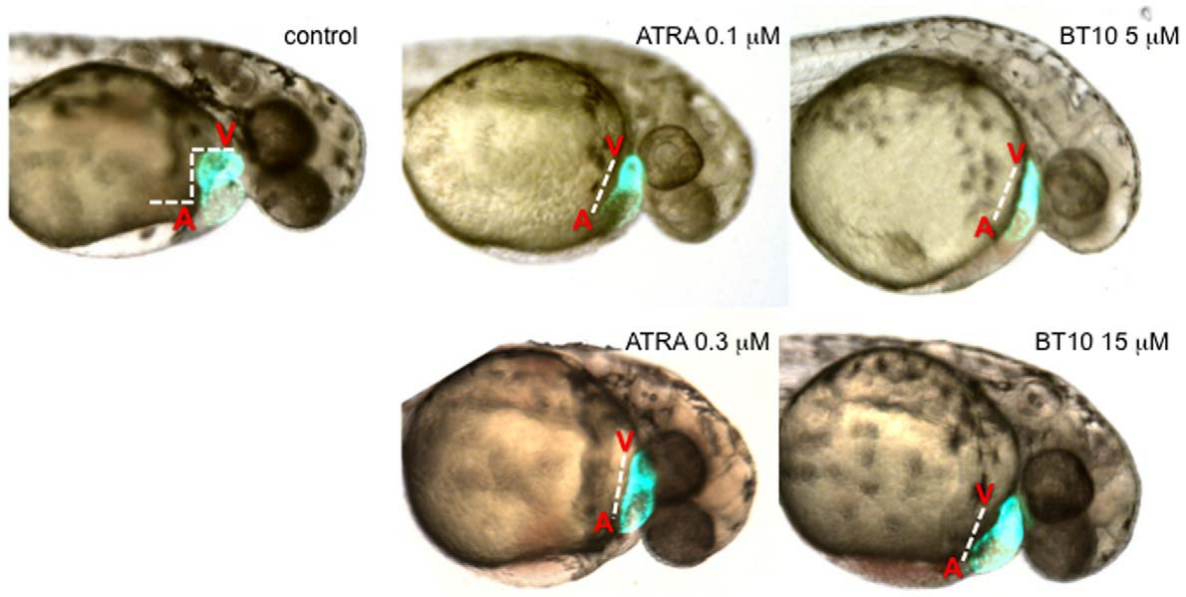
BT10 added at 24 hpf causes a defect in heart tube looping, similar to excess RA. Shown are representative embryos (n = 30) containing a *cmlc2:gfp* transgene were cultured in vehicle alone (control) or in the presence of ATRA or BT10 at the concentrations indicated, and as described in Fig. 3B, beginning at 24 hpf. They were then photographed at 48 hpf in both brightfield and fluorescence, and the images overlayed to document the morphology of the heart tube (green). In control embryos the ventricle (V) is fully looped relative to the atrium (A), indicated by the dotted white line. In contrast, embryos cultured in the presence of ATRA or BT10 develop a relatively linear heart tube (at “low” or “high” concentrations), which is the major obvious morphological abnormality.

We therefore evaluated more closely the phenotype of disrupted heart tube development when similar concentrations of ATRA or BT10 are added later in embryogenesis. For this purpose we exposed embryos derived from transgenic fish carrying a *cmlc2:gfp* reporter, which facilitates visualization of heart tube formation and subsequent cardiac looping (Fig. 6). At these concentrations, heart tube formation occurs normally, and both atrial and ventricular chambers are readily apparent at 48 hpf. However, at 48 hpf the heart remains relatively linear compared to the control vehicle-treated embryos and fails to undergo proper looping morphogenesis. This looping defect likely contributes to the cardiac edema that develops by 60 hpf. Therefore both early and late effects of BT10 on cardiogenesis are consistent with RA-pathway-dependent defects in cell specification or heart tube looping, respectively.

### BT10 is an agonist for retinoid signaling

**Figure 7:**
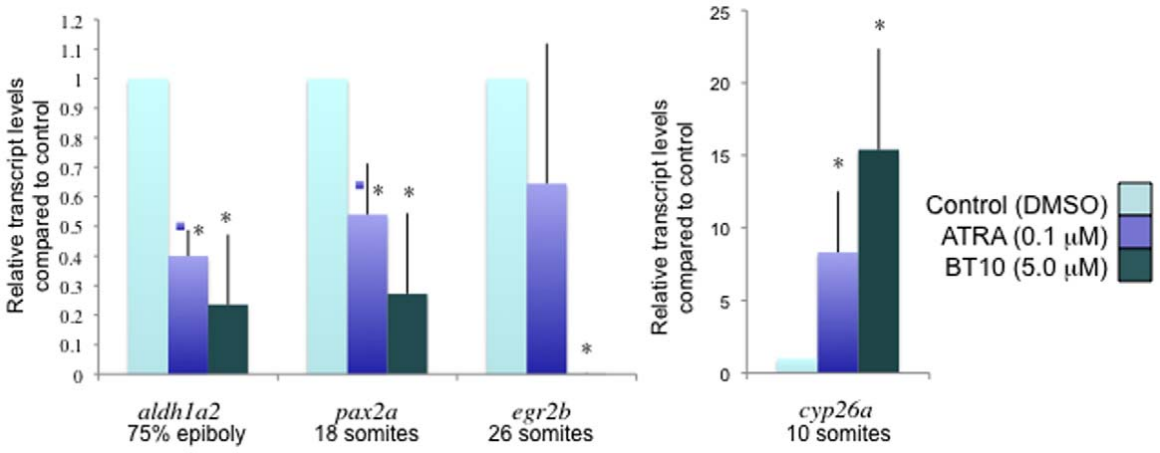
Shown are qPCR results for the markers as indicated, using embryos that were taken at various developmental stages, also indicated. Transcript levels are calculated relative to those from vehicle (DMSO) treated embryos (light blue), for embryos treated with 0.1 µM ATRA (medium blue) or 5 µM BT10 (dark blue). The three markers in the left panel are repressed by both compounds, while *cyp26a* (right panel) is activated. The asterisks indicate statistically significant differences compared to control (p<0.05), based on a Student's T-test.

In order to test directly if BT10 affects retinoid signaling, we analyzed the expression of specific known downstream targets of the pathway when the compound was added to cultured embryos, compared with ATRA or control (DMSO). For this purpose we again used concentrations of the compounds that gave grossly similar body axis defects (5 µM BT10, 0.1 µM ATRA). As shown in Fig. 7, BT10 and ATRA affect target gene expression levels with similar trends, measured by qPCR, consistent for specific activation of the retinoid pathway. For example, both BT10 and ATRA strongly activate expression of *cyp26a1* (a known downstream target that participates in negative feedback), while addition of either leads to significant decreases for genes that are repressed by excessive retinoids, *aldh1a2*, *pax2a*, and *egr2b* (*krox20*).

**Figure 8:**
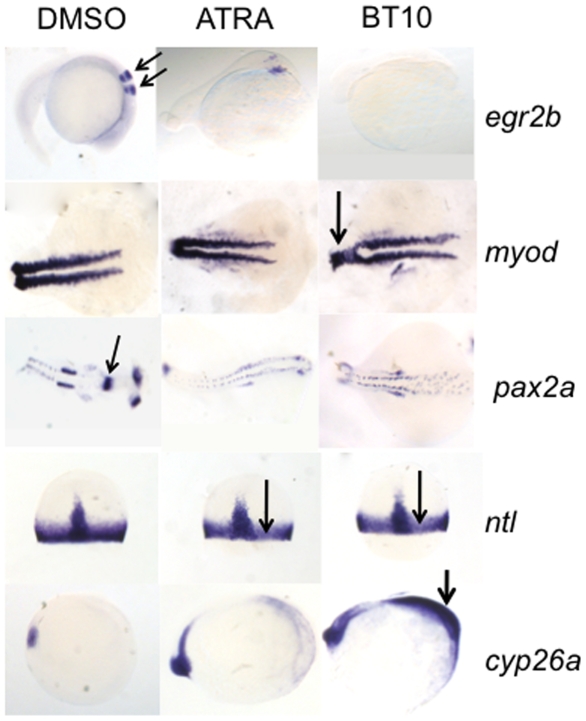
Changes in the expression patterns for known retinoid target genes confirm that BT10 is a retinoid agonist. Shown are representative embryos (n = 30) treated with vehicle (DMSO, left panels), 0.1 µM ATRA (middle panels), or 5 µM BT10 (right panels) and then analyzed by *in situ* hybridization at the 10 somite stage (*cyp26a*), 75% epiboly (*ntl*), the 18 somite stage (*myod* and *pax2a*), or the 26 somite stage (*egr2b*). Embryos are viewed laterally with anterior to the right (*egr2b*, *cyp26a*), dorsally with anterior to the right (*myod*, *pax2a*), or dorsally with animal pole to the top (*ntl*, arrows in both panels). Note that in both ATRA and BT10 treated embryos, the normal anterior expression domains are largely abolished (*egr2b*, arrows indicate normal expression pattern marking the 3^rd^ and 5^th^ rhombomeres in the hindbrain of control embryos, and *pax2a*, arrow indicates normal expression pattern in the midbrain-hindbrain boundary domain), highly reduced (*myod*, disruption noted by arrow in BT10 panel), or more subtly inhibited (*ntl*, indicated by arrows in both panels). In contrast, the expression domain of *cyp26a* is expanded in BT10-treated embryos throughout the anterior region (arrow in BT10 panel).

The specificity of action of BT10 was confirmed by evaluating the spatial expression pattern for retinoid target genes using *in situ* hybridization. As shown in Fig. 8, compared to control, *egr2b* expression in hindbrain segments rhombomere 3 and 5 is eliminated by BT10. Likewise, transcripts for *pax2a* in anterior structures including the midbrain-hindbrain boundary, optic stalk, and otic vesicle, in addition to the ventral pronephric ducts, is largely eliminated. Reduction in the expression domains of *myod* and *ntl* are much more subtle, but consistent with a loss of dorso-anterior mesoderm. The expression domain for *cyp26a1* is expanded throughout the embryo, compared to DMSO-treated embryos in which it is restricted to the posterior tailbud region. BT10 exposure causes a more extensive expansion of this marker compared to ATRA (although note that BT10 is used in this experiment at a much higher concentration compared to ATRA, which is overall more active). Therefore, as suggested by the qPCR data, BT10 appears to function by a mechanism similar but perhaps not identical to ATRA. Both compounds cause similar concentration-dependent changes in marker expression patterns.

### BT10 activity is specific to RAR and not RXR sub-types

**Figure 9:**
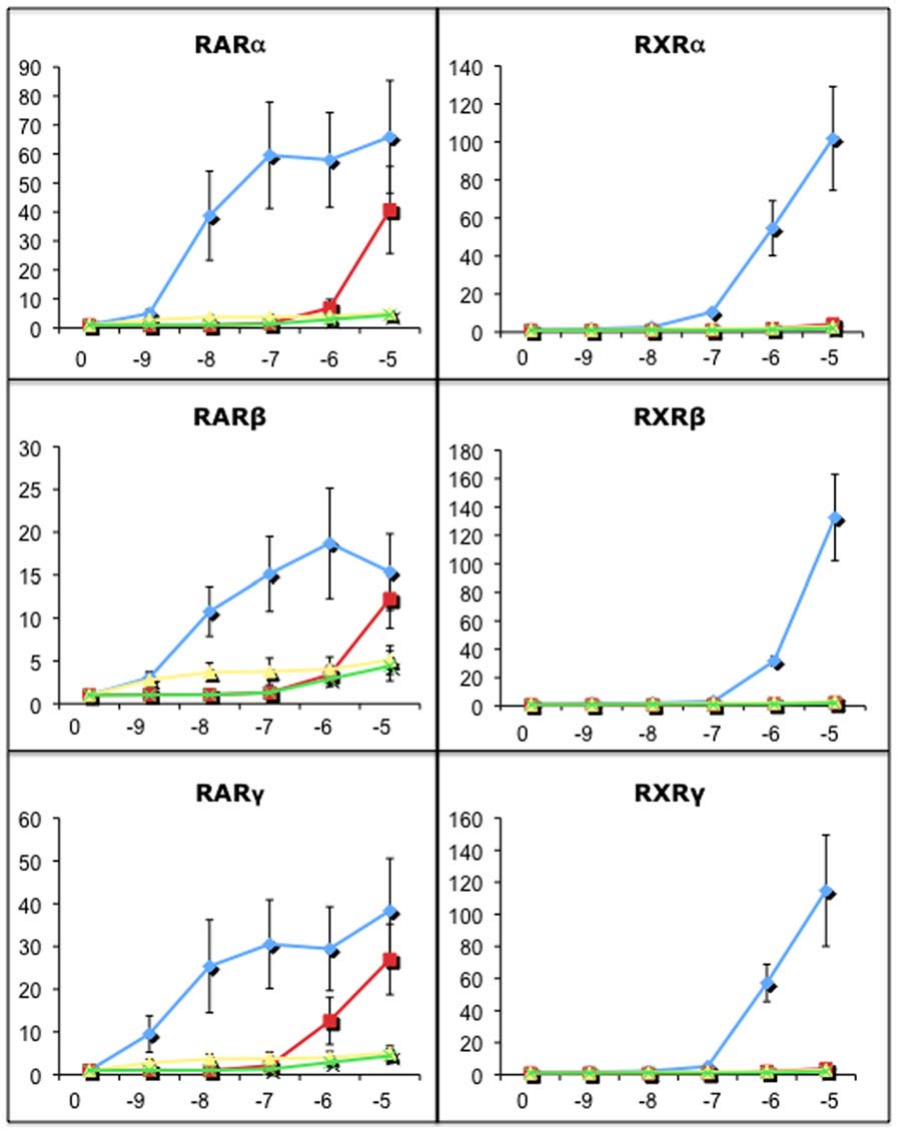
BT10 is selective for RAR receptors. Shown are the results of reporter assays in which specific activated receptors mediate luciferase reporter activity. Hek293T cells were transfected with the indicated RA receptor construct, in addition to the relevant reporter luciferase plasmid, and a non-retinoid regulated renilla reporter plasmid to control for transfection and lysate recovery. Twenty-four hours following transfection, cells were incubated with ATRA (blue diamonds) or BT10 (orange squares), at the doses indicated on the X axis (with the number (x) representing 10^x^ M final concentration). Control transfections lacked the receptor expression construct (yellow triangles or green marked boxes, for ATRA or BT10, respectively). Data is plotted relative to values obtained with transfected cells that were treated with DMSO, with that value set to 1.0, not shown on the graphs. ATRA activates robustly all 6 receptors, while BT10 shows specificity to RARs and does not activate RXRs at all concentrations tested.

**Figure 10:**
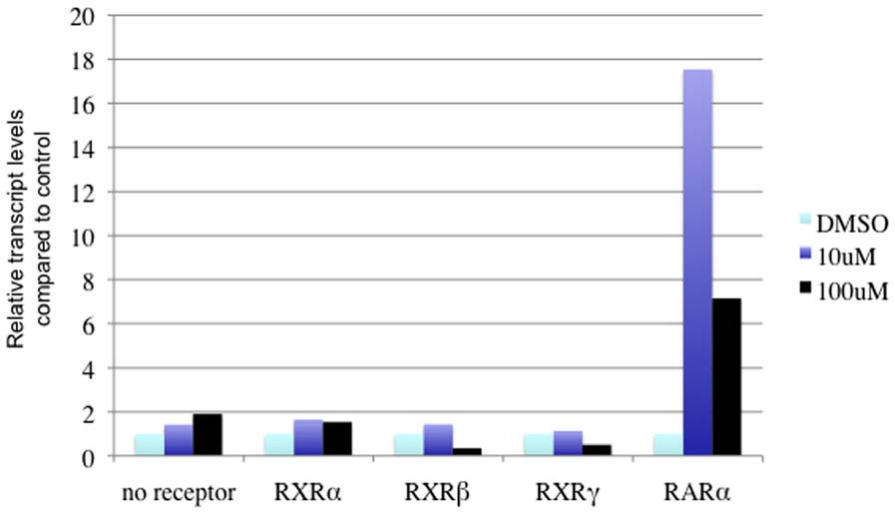
BT10 is selective for RAR receptors even at super-pharmacologic concentrations. Since BT10 has lower apparent affinity to all receptors compared to ATRA, the activity of BT10 was tested at 10 and 100 µM with each RXR isoform. Even at the highest concentration, there is no activation of RXR-dependent reporters, in contrast to activation of the control RARα receptor.

Finally, we performed experiments to test directly if BT10 functions by interaction with retinoid receptors, and we compared its specificity for receptor interaction with ATRA. Retinoids signal through two families of receptors, RAR and RXR, and there are three distinct genes that encode receptors for each family (α, β, and γ). We used a cell culture assay to express receptors and probe the specificity of ligand-receptor interaction [18]. In this assay, the three RARs are fused to the Gal4 DNA-binding domain, and co-transfected with a Gal4-dependent luciferase reporter, and the three RXRs are tested for activation by expression in cells co-transfected with an RA-responsive luciferase reporter. As shown in Fig. 9, ATRA can interact with all 6 receptors, leading to strong activation of the luciferase reporters with a concentration-dependent response. In contrast, BT10 can activate each of the RAR receptors 10–40 fold, but the compound is inactive with respect to RXR activation, at the concentrations tested. Overall, the ability of BT10 to activate the RAR receptors is significantly reduced compared to ATRA, based on the dose response. Activities of the reporters are entirely dependent on co-expressed receptors. Since BT10 has a significantly lower activity for RARs compared to ATRA, we considered that BT10 might have low activity for RXRs, not detected under these conditions. However, even using a 10-fold higher concentration (100 µM) BT10 fails to activate the RXR receptors (Fig. 10). Above this concentration, BT10 is not tolerated by the cultured cells. Therefore, the compound is completely inert with respect to RXR activation, at all concentrations we were able to test. Therefore, BT10 shows interaction with a restricted class of retinoid receptors and appears to target RARs with specificity.

## Discussion

We synthesized a small library of retinoic acid analogues and tested their activity by evaluating phenotypic changes caused in developing zebrafish embryos. Several of the compounds had activity that strongly altered body axis development, including BT10 at concentrations as low as 300 nM. It is interesting to point out the structural similarities and distinctions of the three most active compounds. For compounds 10 and 11 a hydroxyl group is not directly attached to the phenyl ring system but is flanked by mono-methyl (compound 10) or ethyl (compound 11) groups, whereas in compound 20 the phenyl group contains two hydroxyl groups meta to each other. Therefore, it appears that either more hydroxyl groups in the phenyl ring system or a hydroxyl group away from the phenyl ring system makes these retinoids active. It would seem that the three compounds have the same or overlapping targets, since the phenotypes they invoke are essentially indistinguishable according to dose response experiments. However, BT10 appears to have the highest affinity (or at least activity), since it can affect axis development at the lowest concentration. It remains possible that BT10 has enhanced ADME (absorption, distribution, metabolism, or elimination) properties compared to the other compounds. In addition to activity during early embryogenesis, there are targets (the same or different) that continue to be relevant to organ development at later stages, and we documented specific defects in cardiovascular morphology induced between 24–48 hpf. The loss of cardiac tissue when BT10 is added early is likely due to loss of cardiac progenitor tissue, as seen with excess RA. The cardiac morphology defect when BT10 is added at later stages may be indirect, for example due to abnormalities in the vascular or hematopoietic systems. Further studies will be required to understand this phenotype, but it is also seen similarly with excess RA.

Due to the success of differentiation therapy using retinoids to target the PML-RARα oncogene that causes acute promyelocytic leukemia, there has been much interest in the development of retinoids for cancer and metabolic disease therapies. However, retinoids are also teratogens, and there may be significant benefit obtained using ligands with defined and limited receptor specificity (reviewed in [12]). The active target of retinoids is a heterodimer composed of both RAR and RXR receptors. While RAR only interacts with RXR, the RXRs form functional complexes with a variety of additional nuclear receptors. Therefore, pan-agonists may have considerable off-pathway activation (or repression) that might be undesirable or teratogenic. For this purpose, the identification of compounds that are specific to receptor sub-types provide interesting leads of better-defined agonists for differentiation therapy. The gene expression analysis shows that BT10 does affect known targets of ATRA, although there is not a complete concordance with the sensitivity for target gene changes. Based on the morphology dose response study, ATRA is more active than BT10. While BT10 shows stronger effects in the qPCR assays shown here, the relative differences in gene induction or repression may reflect both the different concentrations of the compounds used in the assay, but also receptor complex specificity. This may reflect differential affinity of BT10, showing specificity for RAR and not RXR sub-types. Our results show the utility of combining focused small molecule derivation, applied to a biological screen using the zebrafish model, to identify new compounds with desired target activity and specificity.

## Materials and Methods

### Generation of compounds

All-trans retinoic acid (ATRA) was purchased from Sigma Chemical Co. (Sigma-Aldrich). The dry DMF was stored over 4-Å sieves and degassed before use by bubbling nitrogen through it for at least 1 h. The other reagents and solvents were purchased from commercial sources (Aldrich or Fisher) and used without further purification. All reactions were conducted under a N_2_ atmosphere. The reactions were monitored using TLC (Whatman PE SIL G/UV Fluorescence UV_254_). All the products prepared were purified by flash column chromatography on silica gel grade 62 (60–200 mesh, 150 Å). Proton nuclear magnetic resonance (^1^H-NMR) spectra were recorded in CDCl_3_ using a Bruker 300 MHz instrument. Electrospray Ionization (ESI) mass spectra were determined on a Thermo Finnigan LCQ Classic ion trap mass spectrometer (Waltham, MA) in positive ionization mode.

### Experimental detail of lead compound BT10: (2E,4E,6E,8E)-[3,7-Dimethyl-9-(2,6,6-trimethyl-1-cyclohexenyl)-nona-2,4,6,8-tetraenoylamino]-(4-alcohol)phenylamide

A mixture of all-trans retinoic acid (ATRA) (100 mg, 0.33 mmol) in dry DMF (2 mL) and dry CH_2_Cl_2_ (2 mL) was stirred under nitrogen atmosphere for 1 hr. Oxalyl chloride (1.25 mmol, 110 µL) was added drop by drop at 0°C. The deep red reaction mixture was stirred for another 1.5 h at room temperature under nitrogen atmosphere. After carefully removing the solvent, dry DMF was added (2 mL) immediately. At 0°C under nitrogen atmosphere, Retinoyl chloride solution was added dropwise to a solution of 4-aminobezyl alcohol (0.66 mmol, 81.28 mg) and triethylamine (1.00 mmol, 130 µL) in dry DMF (2 mL). The dark-colored reaction mixture was stirred at room temperature until TLC analysis indicated none remaining (about 2∼3 h). The reaction was quenched with saturated NH_4_Cl and extracted with ethyl acetate. The extracts were washed with H_2_O and brine, then dried overage Na_2_SO_4_, and evaporated. The residue was purified by flash column chromatography using hexane/ethyl acetate (4/1) as the eluent to give BT10 as a yellow solid. 1H-NMR (300 MHz, CDCl_3_): δ 7.58 (d, J = 6 Hz, 2H), 7.34 (d, J = 8 Hz, 2H), 7.28 (s, 1H), 7.12–6.90 (m, 1H), 6.32–6.19 (m, 4H), 5.82 (s, 1H), 4.67 (s, 2H), 2.06–2.03 (b, 4H), 1.74 (s, 3H), 1.67–1.62 (m, 4H), 1.51–1.49 (m, 2H), and 1.05 (s, 6H).^13^C NMR (300 MHz,CDCl_3_): δ166.1, 152.2, 139.3, 138.4, 137.8, 136.7, 131.8, 130.4, 129.3, 128.7, 127.3, 122.8, 120.5, 65.7, 40.1, 34.4, 33.3, 29.0, 22.7, 19.8,1 4.6 and 13.5. HRMS: (C_27_H_35_NO2) Calcd ([M+H^+^]) 406.2746; found 406.2762.

### Screening in zebrafish

For all experiments we used zebrafish that are a hybrid of AB and TU strains. All animal research was conducted according to national and international guidelines, with animals maintained and embryos obtained according to standard fish husbandry protocols [22]. The studies were approved prior to initiating the work by the Institutional Animal Care and Use Committee of Weill Cornell Medical College, and all animals were maintained by trained and approved Animal Institute staff of Weill Cornell Medical College. Embryos were obtained at the one cell stage from paired adults, and cultured in system water. For the initial screen we used embryos at 3 or 5 hpf. They were grouped into individual wells of a 12 well plate, each well containing 3–10 embryos in 1 ml of 1X E3 buffer (5 mM NaCl, 0.17 mM KCl, 0.33 mM CaCl_2_, 0.33 mM MgSO_4_), 1% DMSO, and 1 mM Tris pH 7.5. Compounds were diluted in DMSO to 10 mM stocks, and added to the cultured embryos to a final concentration of 10 µM. Subsequent experiments used further dilutions to determine dose response, and used later staged embryos to define the temporal response.

### Gene expression analysis

Whole-mount *in situ* hybridization was performed essentially as described [23]. Hybridization was performed at 70°C, in 60% formamide buffer with digoxigenin-labeled RNA antisense probes. The probes used for *in situ* hybridization were prepared using either Sp6 or T7 polymerase with linearized templates. Probes were either as described [18], or were first isolated and subcloned by RT-PCR using whole embryo RNA.

For *cyp26a1*, Forward primer: TE2261, TCCGAACTGCAGAAGTCCTC, reverse primer: TE2262, GCTTCCACCAGTTCTTGCTC, product size of 519 bp.

For *egr2b*, Forward primer: TE2265 GCCACTTCTCCAGCACTCTC, reverse primer: TE2266TCTGCGGAGTGTAAGCTGTG, product size of 408 bp.

For quantitative real-time PCR assays, RNA was extracted from staged wildtype or morphant embryos using the RNeasy mini kit (Qiagen). First-strand cDNA synthesis was performed using the Superscript III First-Strand Synthesis System (Invitrogen). The cDNA was analyzed with Qiagen QuantiTect SYBR Green Mix (Qiagen) by quantitative RT–PCR using the Light Cycler 480II (Roche). All samples were prepared in triplicate, and each experiment was repeated at least 3 times using independent batches of embryos. The PCR cycle conditions were 95°C for 15 minutes, (94°C for 15 seconds, 60°C for 30 seconds, and 72°C for 30 seconds) for 40 cycles. The Ct value data were analyzed using the 2^−ΔΔT^ method [24]. Primers used were:

aldh1a2, F: ACTGCCAGGAGAGGTGAAGA, R: CAGGGTTGTACGTGTGGAAG

pax2a, F: GTGTCAAGCGCTTCCAATG, R: CCAGAGCCATCTGAACCATC

egr2b, F: AACACTGCCAGCCTCTGTG, R: GCTTCTCCGTGCTCATATCC

cyp26a, F: TCCTGGAGGATTCAGAGTCG, R: ATCGTGCGTGTCACAGATG

### Receptor specificity assays

Hek293T cells were grown in DMEM containing 10% fetal bovine serum for 24 hr and 5×10^4^ cells were transfected with 200 ng pCMX-Gal-L-hRAR, 50 ng tk-px3-luc, and 1 ng renilla luciferase reporter plasmid or 200 ng pCMX-mRXR, 50 ng tk-apoA1-luc and 1 ng renilla plasmid, using Lipofetamine 2000, according to the manufacturer's protocol (Invitrogen). Control transfections lacking receptors included only 50 ng tk-px3-luc or tk-apoA1-luc and 1 ng renilla luciferase reporter plasmid. Twenty-four hours following transfection, cells were treated with various concentrations (10^−9^ M to 10^−3^ M) of ATRA or BT10 diluted into DMSO, or were treated with DMSO alone. Cell lysates were collected 48 hr after transfection and were assayed for luciferase activity using the Dual-Luciferase® Reporter Assay System (Promega). The luciferase values were normalized to the renilla luciferase reporter, and results further normalized to the values obtained in cells treated with vehicle (DMSO) alone. The results were obtained from two independent experiments in assays done each time in duplicate.
